# Identification and characterization of abundant repetitive sequences in *Allium cepa*

**DOI:** 10.1038/s41598-019-52995-9

**Published:** 2019-11-14

**Authors:** Jiaping Fu, Hao Zhang, Fengling Guo, Lu Ma, Jinping Wu, Mengxia Yue, Xueke Zheng, Zhengming Qiu, Lijia Li

**Affiliations:** 1 Institute of Economic Crops, Hubei Academy of Agriculture Sciences, Wuhan, 430064 China; 2 State Key Laboratory of Hybrid Rice, College of Life Sciences, Wuhan University, Wuhan, 430072 China; 3 Shenzhen Tobeacon Technology Co. Ltd., Shenzhen, 518049 China

**Keywords:** Plant sciences, Genome duplication

## Abstract

Species of the genus *Allium* are well known for their large genomes. *Allium cepa* is of great economic significance. Among vegetables, it ranks second after tomato in terms of the global production value. However, there is limited genomics information available on *A*. *cepa*. In this study, we sequenced the *A. cepa* genome at low-coverage and annotated repetitive sequences by using a combination of next-generation sequencing (NGS) and bioinformatics tools. Nearly 92% of 16 Gb haploid onion genome were defined as repetitive sequences, organized in 162 clusters of at least 0.01 percent of the genome. Of these, a proportion representing 40.5% of the genome were further analyzed in detail to obtain an overview of representative repetitive elements present in the *A. cepa* genome. Few representative satellite repeats were studied by fluorescence *in situ* hybridization (FISH) and southern blotting. These results provided a basis for evolutionary cytogenomics within the *Allium* genus.

## Introduction

The C value, which is the DNA content in a gamete, may vary widely among closely related organisms. Large comparative analyses of the plant DNA C-value database (http://data.kew.org/cvalues/) have shown great variation in terms of genome size in plants^1^. Part of this variation is due to differences in the ploidy level, while the majority of the variation is based on differences in the repeats. There is a lack of correlation between genome size and complexity of eukaryotic genomes, which is called C-value paradox. The main reason for genome size variations are differences in the abundance of transposable elements (TEs), while gene numbers remain relatively constant^2^.

The genus *Allium* (Amaryllidaceae) belongs to monocotyledonous genera and includes more than 800 species, which are widely distributed over the Holarctic region from the dry subtropics to the boreal zone^3^. This genus includes the most common chromosome number x = 8 and other numbers (e.g., x = 7, 9, 10, 11) and a number of polyploids are characterized by a large genome size. The genome size of 30 *Allium* species varies from 7 pg (*A. altyncolicum*, 2n = 4 ×  = 32) to 31.5 pg (*A. ursinum*, 2n = 2 ×  = 14)^4^. The genus *Allium* is of great economic importance because it includes several important vegetable crops and ornamental species, such as *A. cepa* L. (onion), *A*. *sativum* (garlic), *A. schoenoprasum* (chives), *A. wakegi* (scallion), *A. cepa* var. aggregatum (shallot), and *A. ampeloprasum* var. porrum (leek). In terms of the global production value, onion ranks second after tomato. While the tomato genome is fully sequenced, little is known about the onion genome. This is partly attributed to the giant size of the onion genome (1 C = 16 Gbp), which consists of more than 95% of repetitive elements^5^.

An increasing number of plant genomes are fully sequenced (https://genomevolution.org/wiki/index.php/Sequenced_plant_genomes). But it is still a challenge and costly to sequence large genomes such as, that of *A. cepa*. Therefore, in the onion genome sequencing project (SEQUON, http://www.oniongenome.net), it is proposed to only sequence gene-rich regions combining transcriptome sequencing and enrichment of low-copy regions by removing repetitive DNAs. Until now, only reference gene sets have been revealed by transcriptome sequencing^6^. The study of repetitive sequences in onion has mainly focused on tandem repeats, including the 375 bp satellite sequence family^7–9^ and telomeric repeats^10^. Mancia *et al*. (2015) analyzed the distribution of rDNA, cot-1 DNA and 375 bp satellite in two *A. cepa* varieties by FISH^9^. There was no comprehensive analysis of repetitive sequences in *A. cepa*. Until recently, a comparative study of the repeatome on three *Allium* species, including onion has been performed^11^.

Based on advances in next-generation sequencing (NGS), *de novo* assembly and annotation methods, repetitive sequences can be studied effectively at reasonable costs by combining low-pass NGS^12^ and graph-based clustering analysis^13,14^. Differences in genome sizes between *Arabidopsis* (1 C = 150 Mb) and *A. cepa* (1 C = 16 Gb) are mainly due to the amplification of repetitive DNA sequences. Repetitive sequences include tandem repeats (satellites, minisatellites and microsatellites) and transposable elements (TEs)^15^. As in all other eukaryotes, TEs in plants are categorized as class I (retrotransposons) or class II (DNA transposons) transposons. Class I (retrotransposons) contains all TEs that transpose via an RNA intermediate in a “copy-and-paste” process. Class II DNA transposons transpose through a DNA intermediate via a “cut-and-paste” mechanism, usually maintaining a moderate copy number in the genome^16^. In this study, we sequenced the *A. cepa* genome at low-coverage, identified and characterized its most abundant repetitive sequences and determined the chromosomal localization of a few repeats.

## Materials and Methods

### Materials

*A. cepa* cv. Fuxing was obtained from Institute of Economic Crops, Hubei Academy of Agriculture Sciences, China.

### DNA extraction for NGS

We collected leaves and roots from the greenhouse grown seedlings and carried out DNA extraction using a DNeasy plant mini kit from Qiagen.

### NGS

A sequencing library was prepared using NEB Next® Ultra™ DNA Library Prep Kit Illumina (New England, Biolabs, Ipswich, MA, USA). Paired-end sequencing (2 × 150 bp, 350–400 bp insert size) of total genomic DNA was performed by iGeneTech Co. Ltd. (Beijing, China) on the Illumina HiSeq2500 platform on a single lane. Clean sequencing data were supplied in FASTQ format without adapters.

### Repeat explorer

The RepeatExplorer pipeline^14^ (https://repeatexplorer-elixir.cerit-sc.cz/galaxy/) was performed to cluster NGS raw reads into groups of similar reads with default setting. Repeat clusters with genome proportions of no less than 0.01% were selected for further analysis. Repeat clusters with known protein domains can be classified by RepeatExplorer pipeline directly. Other clusters were subjected to manual analysis with similarity searches against GenBank databases (Nt and Nr) using Blastn and Blastx^17^ with an E-value of 1e^−5^.

### PCR amplification, cloning, and sequencing of AceSat01–377 and AceSat02–750

Amplification of the determined repeats was performed by using specific primers (Table 1) designed by NCBI Primer blast (https://www.ncbi.nlm.nih.gov/tools/primer-blast). The PCR conditions were 98 °C 1 min, 35 cycles: 98 °C 15 s; 60 °C 15 s; 72 °C 30 s; final elongation: 72 °C 3 min. The sequences of the repeats were validated by cloning of the PCR product into pGEM-TEasy vector (Promega, Madison, WI, USA) according to manufacturer’s instruction. The individual clones were sequenced using an ABI 3130xl Genetic Analyzer. The sequences of the repeat units were submitted to NCBI GenBank (Table 1).

**Table 1:** Summary of the tandem repeats.

Tandem repeats	Primers 5′–3′	Accession number in NCBI	Size of repeat unit (bp)	Expected length of PCR product (bp)
AceSat02–750	F:TCACACTgTAgCACTCgATATTAAAg	MH017541	750	802
	R:TTTATTCCgTCggTgATCCA			
AceSat01–377	F: gATgTTgCATCATCCACACg	MH017542	377	610
	R: ggTgTCgAAAAAAATgAAggg			

### Chromosome preparation

The chromosomes are prepared as mentioned by He *et al*.^18^. Root tips of *A. cepa* cv. Fuxing were collected when they reached 0.5–1 cm. Mitosis was blocked in α- bromonaphthalene avoiding light at room temperature for 4 hours, followed by fixing in 4% (w/v) paraformaldehyde, stored at 4 °C for 40 min. After that the root tips were digested by 2% cellulase and 2% photolyase for 60 min at 37 °C. The digested root tips were homogenized in 60% acetic acid solution and dripped on glass slides. The prepared slides were dehydrated with an ethylalcohol series.

### Fluorescence *in situ* hybridization (FISH)

pTa71 of 45 S rDNA was used in the present investigation. The repeated DNA sequences used for FISH were labelled by nick translation with RED: Texas Red-12-dUTP (Invitrogen C3176) and GREEN: Fluor 488–5-dUTP (Invitrogen C11397) FISH was performed as described^18^. The slides were treated in 70% formamide in 2 × saline sodium citrate (SSC) for 5 min at 90 °C. Simultaneously, 2 mg/ml probes and 1 mg/ml sheared salmon sperm DNA were pre-mixed in 2 × SSC with 10% dextran sulphate and 50% deionized formamide, denatured at 80 °C for 5 min and immediately cooled in ice water. After dehydrating and air drying, the slides were incubated in denatured hybridization solution overnight at 37 °C. Nuclei and chromosomes were stained with 4, 6- diamidino-2-phenylindole (DAPI, 0.2 mg/ml, Sigma, Deisenhofen, Germany) and observed under an Olympus BX-60 fluorescence microscope. Images obtained using a CCD monochrome camera Sensys 1401E were pseudo-colored and processed with the Metamorph imaging system (Universal Imaging Corp., PA, USA. version 4.6.3) and Adobe Photoshop 9.0 software.

### Southern blotting

Genomic DNA was digested by restriction enzymes *EcoRI* and *XbaI* respectively at 37 °C for 12 h. Southern blotting was carried out using the method described by Li *et al*.^19^. Nylon transfer membranes were MILLIPORE.IMMOBILON-NY + 30 cm × 3.3 m Roll (Catalog No. INYC00010). Repeated DNA sequences used for southern blotting were labelled by nick translation with digoxin-11-dUTP (Roche, Mannheim, Germany). Hybridization was performed using the DIG High Primer DNA Labeling and Detection Starter Kit I (Cat.No.11 745 832 910, Roche Diagnostics GmbH, Germany). Detection was performed using alkaline phosphatase (AP) conjugated anti-DIG antibody (1:5000) and chemiluminescence visualization with 5-bromo-4-chloro-3-indolyl phosphate/ nitro blue tetrazolium (BCIP/NBT).

## Results and Discussion

### Genomic repeatome composition

**Figure 1:**
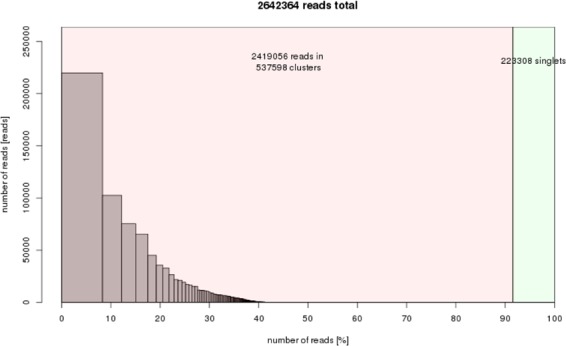
Repeat composition of clusters generated by RepeatExplorer of 2,642,364 reads (2.16% of genome coverage). And the RepeatExplorer pipeline arranged 2,419,056 reads in 537,598 clusters. The genomic proportion of these identified repeats was about 92%. X-axis: cumulative proportion of clusters of the genome. Y-axis: numbers of reads.

Because of its large genome size of about 16 Gbp/1 C, it is difficult to analyze the repeat composition of the whole onion genome by traditional molecular methods^20^. Thus, we used the latest NGS technology and RepeatExplorer computational pipeline to reveal the genome structure. A total of 15,990,607 clean paired-end reads were obtained with a length of 150 bp each. Illumina sequencing can lead to a bias at the beginning and the end of reads^21^. Therefore, we trimmed the 10 bp on both ends of each read. Reads with quality score >  = 10 over 95% of bases without Ns were further analyzed. Only complete read pairs (interlaced reads) were used. The RepeatExplorer pipeline revealed us to use 0.01–0.5 × genome coverage reads for analysis (https://repeatexplorer-elixir.cerit-sc.cz). To maintain a balance between high sensitivity and moderate running times with the available computational resources, we decided to use 2,642,364 reads representing ca. 2.16% of the genome for clustering in RepeatExplorer pipeline. The RepeatExplorer pipeline arranged 2,419,056 reads in 537,598 clusters, and nearly 92% of the genome were found to be repetitive sequences (Fig. 1). Top 162 clusters of not less than 0.01% of the genome comprised ~40.5% of the genome. The total repeat composition is similar as previously reported^5^. Until now, only a few giant genomes have been analyzed for repetitive DNA composition; most of them are composed of highly heterogeneous groups with relatively low abundance of repeat-derived DNA. For example, in the Australian lungfish (*Neoceratodus forsteri*) genome (~50 Gbp/1 C), only 40.2% can be assigned to recognizable repetitive DNA^22^; in the black salamander (*Aneides flavipunctatus*) with a genome of ~44 Gb, less than 50% can be assigned to known TEs^23^. The genome size of diploid *Fritillaria* species varies between 30.15 and 85.38 Gb; about 42% of the genome was assigned to known TEs in *F. imperialis*^24^. Our results suggested that unlike other giant genomes, the genome of *A. cepa* seems to be more similar to smaller genomes because very large genomes are usually derived from massive amplification of a small number of LTR retrotransposons^25^. The 162 clusters of at least 0.01 percent of the genome represent together 40.5% of the genome and were further annotated (Fig. 1 and Table 2). No coding genes were found in these clusters except rDNA, mobile elements and plastid genes (ca. 1.05%). The proportion of repeat types within the *A. cepa* genome was shown (Table 2). The most abundant repeats are LTR-retrotransposons, including 14.227% Gypsy and 3.569% Copia elements. The genome also consists of 8.599% of low complexity repeats and 8.393% of unknown repeats, which may be due to lack of sufficient annotated sequences from close related species in public database. In addition, there were 1.912% of simple sequence repeats (4 clusters), 1.421% of satellite sequences (3 clusters), 0.581% of rDNA (1 cluster), 0.528% of DNA. CMC.EnSpm (3 clusters) and 0.22% of LINE.L1 repeats (1 cluster). There are ca. 50.049% of the tiny repeat clusters composing ca. 8 Gb DNA sequences in *A. cepa*, which stay undetermined due to the RepeatExplorer threshold for clustering. The question if they come from the mainstream repeats as degenerative copies or if they are unrelated to them stays to be answered. Hertweck and Bainard used *de novo* repeat assembly methods (MSR-CA) rather than graph-based clustering methods (RepeatExplorer) and assembled *Allium fistulosum* repeats by low coverage single-end reads. And annotated repeats are about 9% of the genome, which are likely underestimated^26^. Kiseleva *et al*. (2014) applied RepeatExplorer by 10,725 genome survey sequences around 1,000 bp each of *A*. *cepa* with focus on centromeric Ty3/gypsy retrotransposons^27^. Peška *et al*. (2019) analyzed repeatome of three *Allium* species (*A*. *cepa*, *A*. *sativum*, and *A*. *ursinum*) and defined 60% of genome of *A*. *cepa* represented by repetitive sequences 11, which is much lower than the present findings (ca. 92%). We selected 600,000 reads from *A. cepa* NGS data released by Peška *et al*. (2019) and did the co-clustering with 600,000 reads from current study. And we confirmed the difference came from these two datasets themselves rather than personal errors between different laboratories (Fig. S6). However, Peška *et al*. (2019) used PCR free library for NGS, while we used normal library for sequencing. In addition, the difference might be attributed to intraspecific variability caused by different cultivars used in both studies.

**Table 2:** Repeat composition of *Allium cepa* genome estimated from the Illumina sequencing data.

Repeat type	Genome proportion (%)
Low_complexity	8.599
Simple_repeat	1.912
Satellite	1.421
rDNA	0.581
DNA.CMC.EnSpm	0.528
LINE.L1	0.22
Plastid	1.05
Unknown in analyzed clusters	8.393
Total in analyzed clusters	40.5
Small clusters that were not analyzed	51.049
Non-clustered reads	8.451
Total	100

### PCR amplification, cloning and sequence analysis of satellite DNAs

Tandem repetitive DNA is an important component of the repetitive sequences. According to the length of the repeat arrays and their sizes, tandem repetitive DNA sequences can be classified into three groups: (i) microsatellites with 2–5 bp repeats and an array size of the order of 10–100 units, (ii) minisatellites with 6–100 bp (usually around 15 bp) repeats and an array size of 0.5–30 kb and (iii) satellite DNA (satDNA) with a variable AT-rich repeat unit that often forms arrays up to 100 Mb, suitable as chromosome markers in FISH experiments^8,28^.

**Figure 2:**
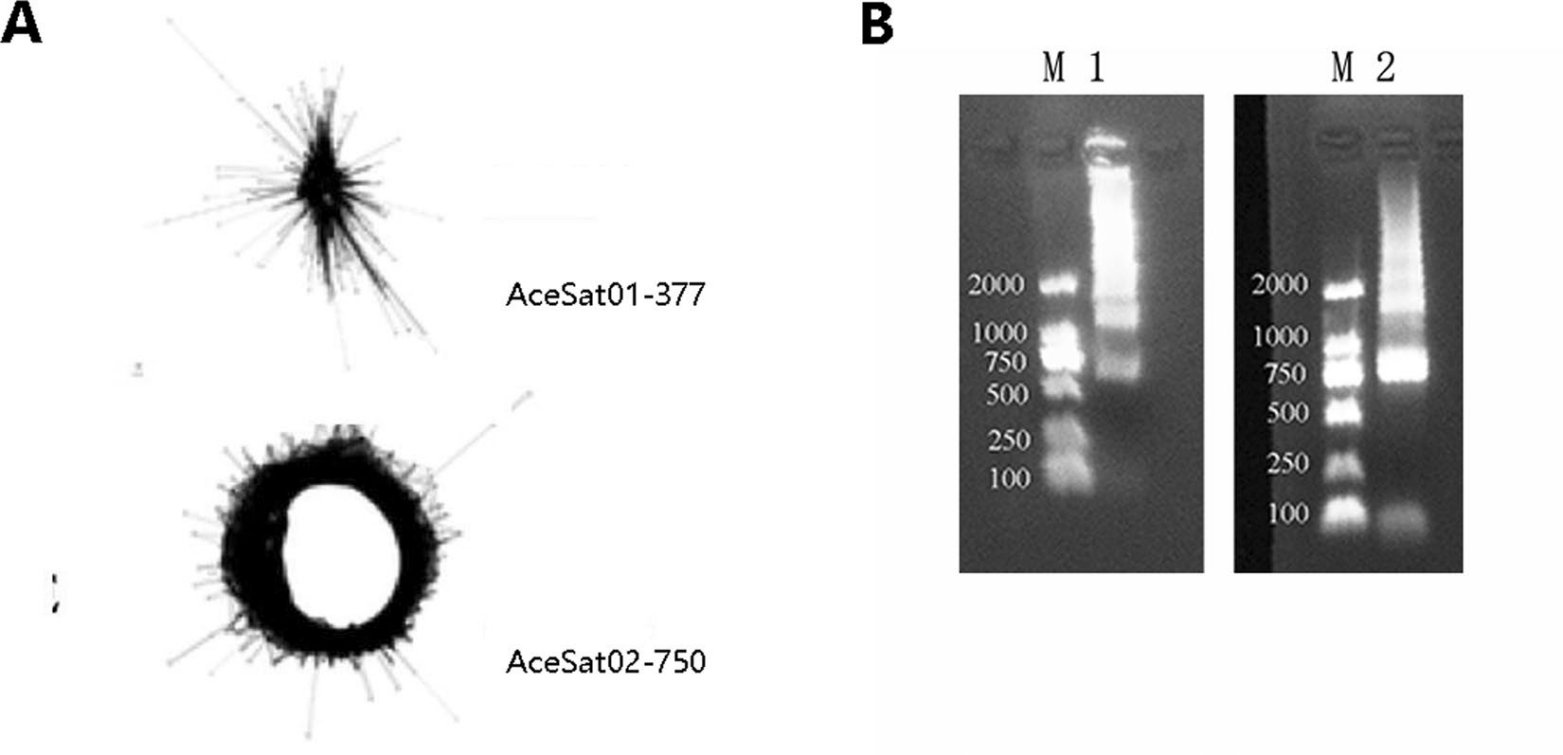
(**A**) The layout of AceSat01–377 and AceSat02–750 clusters from RepeatExplorer. (**B**) PCR amplification with primers for AceSat01–377 (1) and AceSat02–750 (2) repeats. M: marker.

NGS and graph-based clustering analyses provide high-throughput tools for detecting satellite DNA^28,29^. As suggested by Ruiz-Ruano *et al*.^29^, the satDNA terminology should begin with species abbreviation in Repbase (e.g. Ace for *Allium cepa*) followed by the term “Sat”, a catalog number in order of decreasing abundance (according to the first genome analyzed), followed by consensus monomer length. Therefore, we termed two satellites as AceSat01–377 for cluster 7 (AcCL7) and AceSat02–750 for cluster 43 (AcCL43). Such satellite DNA usually showed a globula-like (AceSat01–377) or ring-like (AceSat02–750) graph^30,31^ (Fig. 2A). The AceSat01–377 and AceSat02–750 clusters comprised of 1.24% and 0.17% of the genome, respectively. The monomer length of satDNA sequences ranges from 150–400 bp in majority of plants and animals^15^. The length of the monomers for AceSat01–377 and AceSat02–750 are 377 bp and 750 bp, respectively (Table 1). These two repeats have been cloned and sequenced (GenBank accession numbers are MH017542 for AceSat01–377 and MH017541 for AceSat02–750). AceSat01–377 showed high similarity with a satellite commonly found in *Allium* species^32^ and low diversity among monomers. For AceSat02–750, BLASTn analysis revealed no significant match against National Center for Biotechnology Information (NCBI) databases (e–value = 10^−5^), suggesting that this might be a novel satellite repeat.

**Figure 3:**
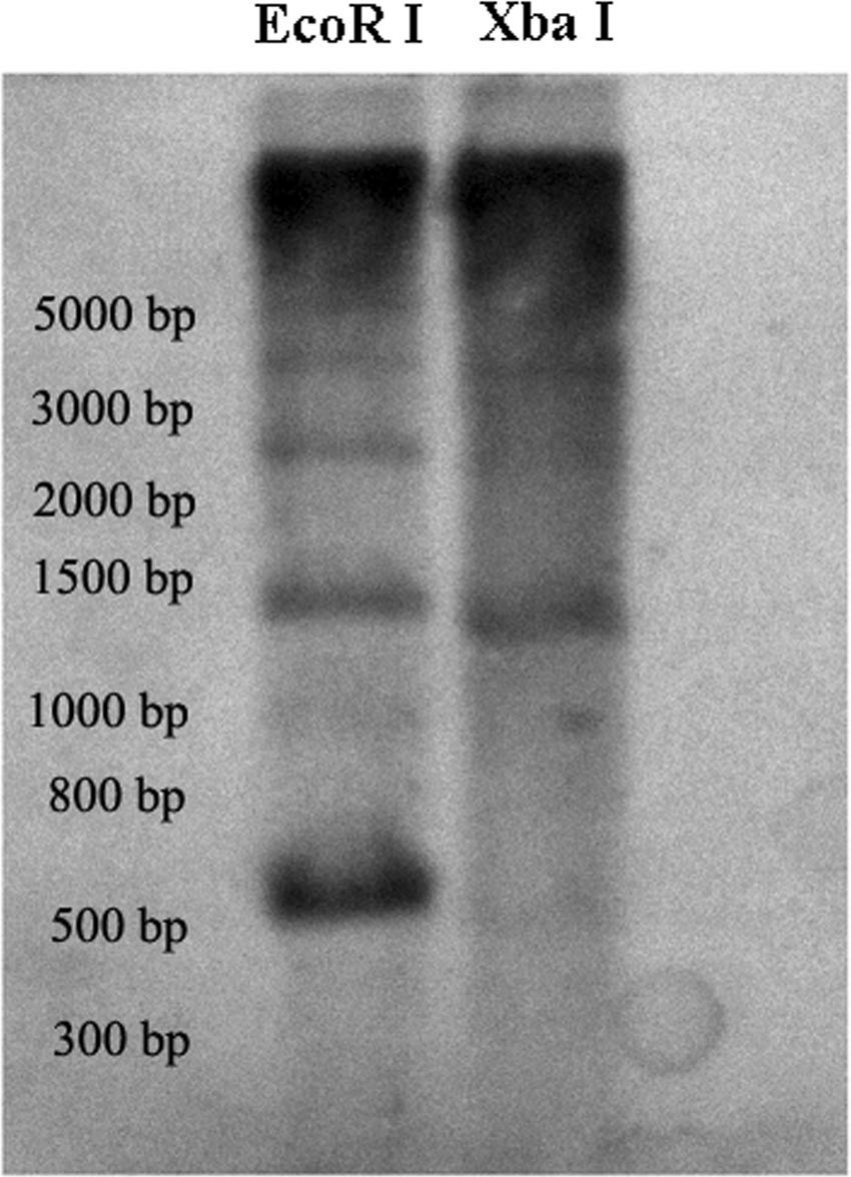
Southern blotting analysis of AceSat02–750 revealed a ladder-like pattern typical for satellite DNA. The genomic DNA was digested with *EcoRI* and *XbaI* separately.

PCR with AceSat01–377 and AceSat02–750 primers with genomic DNA of *A. cepa* resulted in ladder-like PCR products (Fig. 2B, S2 and S3), confirming the tandem organization of these repeats in *A. cepa*. The AceSat02–750 clone was used as a probe for southern hybridization of genomic DNA. After *EcoRI* and *XbaI* digestion, AceSat02–750 revealed a ladder-like pattern (Fig. 3 and S3B), confirming its organization in the form of tandemly repetitive sequences.

### Chromosome localization of AceSat01–377 and AceSat02–750

**Figure 4:**
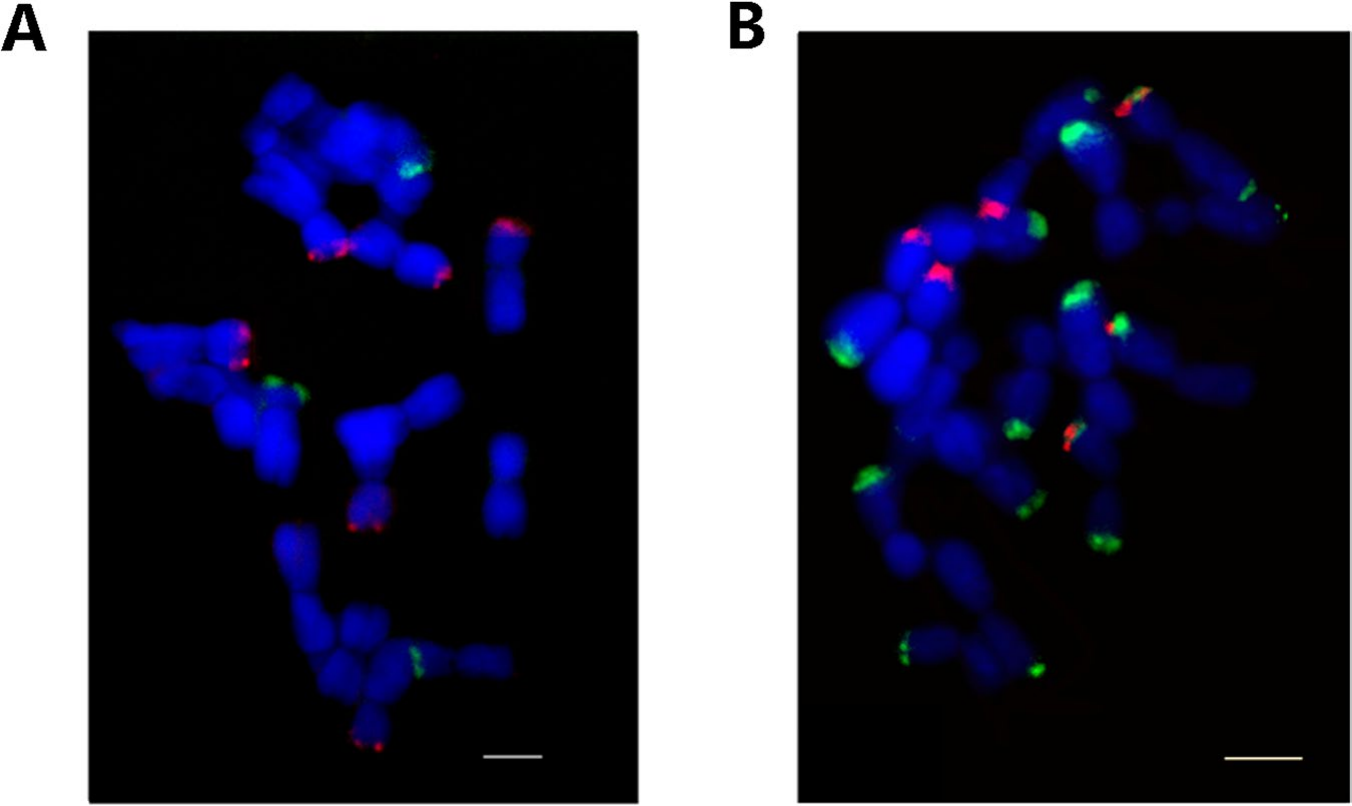
FISH with repeats on *A. cepa* chromosomes. (**A**) 45 S rDNA (green) and AceSat02–750 (red). (**B**) AceSat01–377 (green) and AceSat02–750 (red). Scale bar = 10 um.

**Figure 5:**
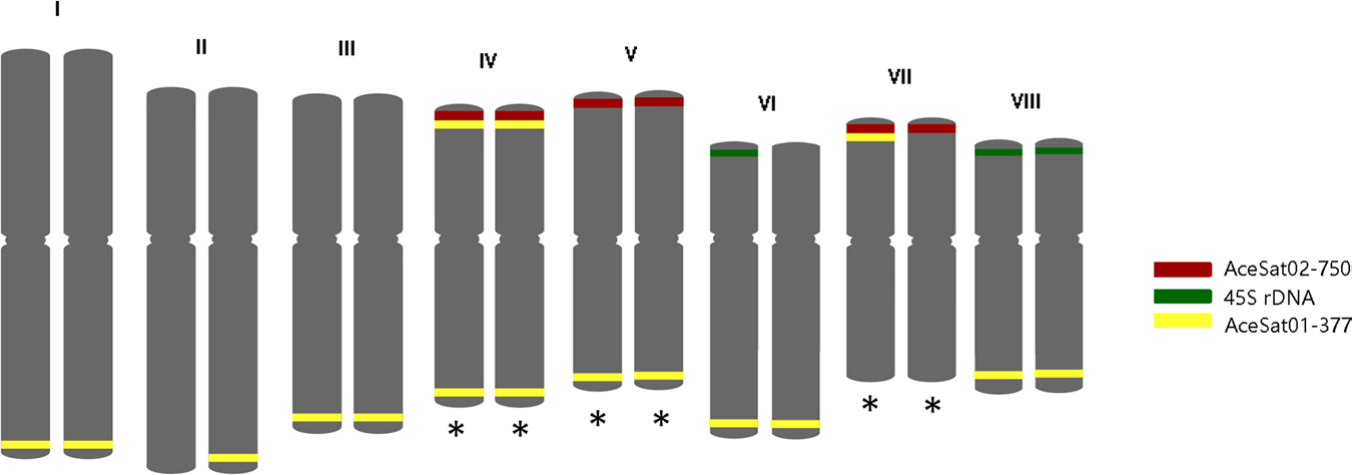
Idiograms of *A*. *cepa* chromosomes with marked localization of AceSat01–377 (yellow), AceSat02–750 (red) and 45 S rDNA (green) repeats. The chromosomes which could be identified are marked with*.

SatDNA sequences were located at heterochromatic regions, which appear not only at the centromeric and subtelomeric regions of the chromosomes, but also at intercalary positions. FISH with AceSat01–377, AceSat02–750 and 45 S rDNA was carried out on the metaphase chromosomes of *A. cepa* to investigate their chromosomal distribution (Figs 4 and 5). 45 S rDNA localized on three chromosomes (Fig. 4A and S5). In most cases in *A. cepa*, there are two pairs 45 S rDNA loci reported^8^. However, in *A. cepa*, two, three, or four loci of 45 S rDNA would be expected, which might be due to mobility of NOR^33^. The AceSat01–377 clone hybridized at sub-terminal regions at both ends of only one pair of chromosomes, and labeled only one end of rest of chromosomes except two chromosomes (Figs 4B and 5). The heterozygous signals of AceSat01–377 for each chromosome are strong enough and it is unlikely one chromosome is unlabeled while the homologous is labeled due to technique issue. In addition, the heterozygous of rDNA and repeats are reported in *Allium cepa*^33^, *Vicia faba*^34^ and many other plants. AceSat02–750 hybridized to sub-terminal regions on three pairs of chromosomes. AceSat01–377 and AceSat02–750 are co-localized on three chromosome arms (Figs 4B and 5). Peška *et al*. (2019) also analyzed distribution pattern of similar repeats on chromosomes by FISH^11^. The AcepSAT356 and AcepSAT750 are similar to present AceSat01–377 and AceSat02–750, respectively, but the differences of FISH pattern in these two studies suggested the cultivar difference of these two repeats in *A. cepa*. AceSat02–750 occurred distal to AceSat01–377 at one end of three chromosomes (Figs 4B and 5). Taking together, we could identify 6 out of 16 chromosomes in *A. cepa* combining these three probes (Fig. 5). However, we failed to get FISH signals with other candidates (Table S1). Possibly, they are clustered in small groups, which are not sufficient to yield unambiguous FISH signals.

## Supplementary information

FASTA Sequence decument DNA

Supplementary Information

## Data Availability

All the data pertaining to the present study have been included in tables and/or figures in the present manuscript and the raw reads of sequencing data have been uploaded on the NCBI SRA database. The output of RepeatExplorer archive has been uploaded on the Figshare.com (https://figshare.com/s/b5adf97d66269b0369bc).
