# Supplementary material for: Identification and characterization of abundant repetitive sequences in Allium cepa
Source: Sci Rep. 2019 Nov 14;9:16756. doi: 10.1038/s41598-019-52995-9 (PMC6856378; doi:10.1038/s41598-019-52995-9)
Supplement: Supplementary file 2 — Supplementary Information [file 41598_2019_52995_MOESM2_ESM.docx]

**Identification and characterization of abundant repetitive sequences in *Allium cepa***

Jiaping Fu^1#^, Hao Zhang^2#^, Fengling Guo^1^, Lu Ma^3^, Jinping Wu^1^, Mengxia Yue^2^, Xueke Zheng^2^, Zhengming Qiu^1^*, Lijia Li ^2^*

1. Institute of Economic Crops, Hubei Academy of Agriculture Sciences, Wuhan 430064, China

2. State Key Laboratory of Hybrid Rice, College of Life Sciences, Wuhan University, Wuhan 430072, China

3. Shenzhen Tobeacon Technology Co. Ltd., Shenzhen, 518049, China

*Correspondence author email: [ljli@whu.edu.cn,](mailto:ljli@whu.edu.cn) [13808640602@163.com](mailto:13808640602@163.com)

#: Jiaping Fu and Hao Zhang contributed equally to this work.

Email addresses of authors:

Jiaping Fu fujiaping2009@163.com

Hao Zhang 2009301060071@whu.edu.cn

Fengling Guo guofenglingok@163.com

Lu Ma 42602510@qq.com

Jinping Wu 18971632353@163.com

Mengxia Yue 1806117293@qq.com

Xueke Zheng 1261110509@qq.com

Zhengming Qiu 13808640602@163.com

Lijia Li ljli@whu.edu.cn


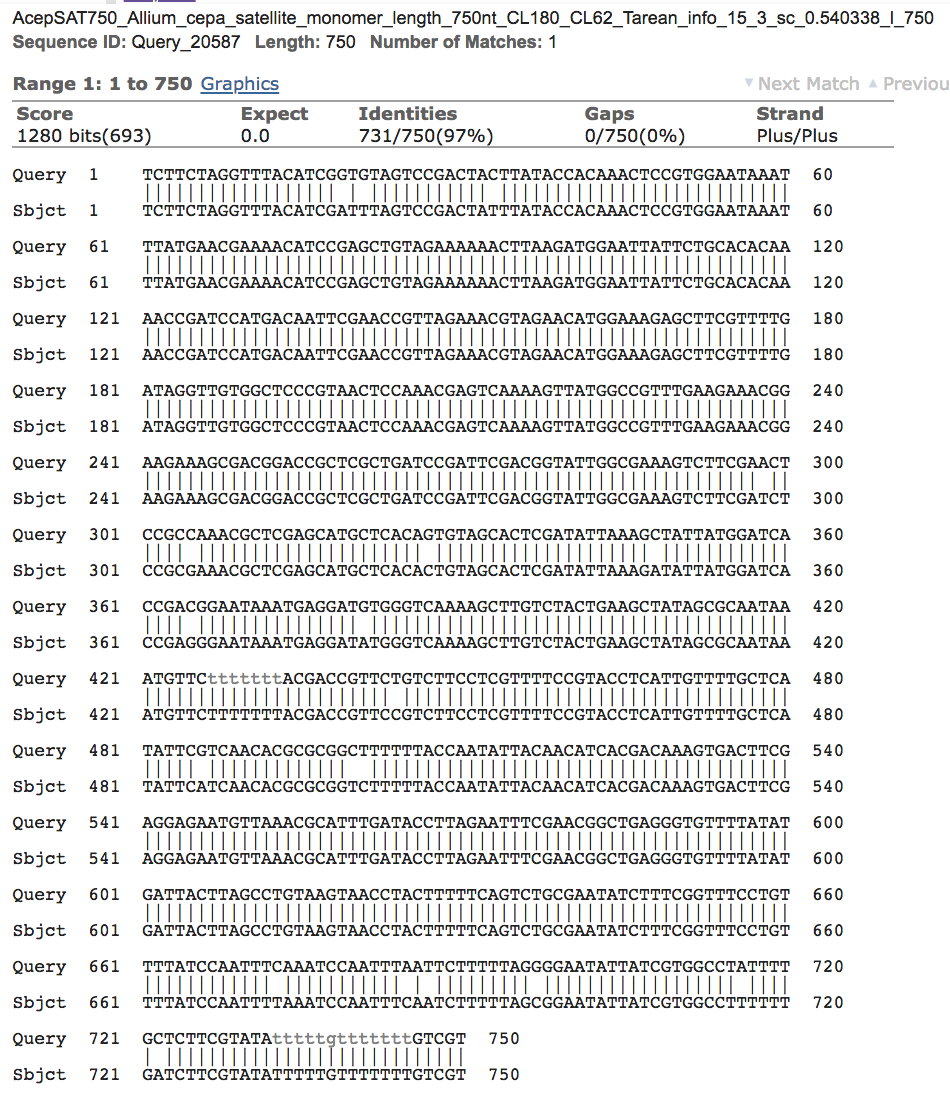


Figure S1 Alignment of the 750bp sequence from Peska et al. 2019 and AceSat02-750 from this study. There is 97.52% (731/750) identity between these two sequences.


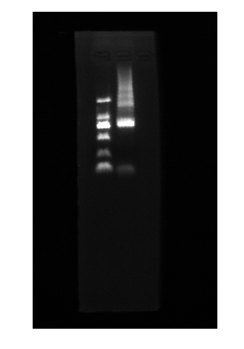


Figure S2 A raw image for PCR application of the AceSat02-750 DNA. The left line is a DNA size marker.


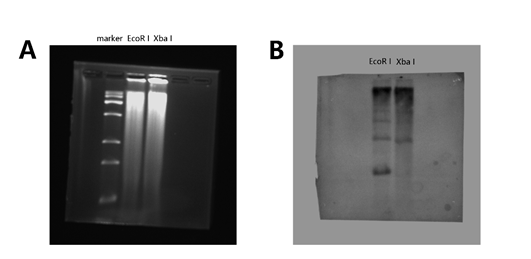


Figure S3 Raw figures for southern hybridization.

1. Genomic DNA was digested with EcoR I and Xba I. The DNA size markers are shown on the left.
2. Southern hybridization of genomic DNA with the AceSat02-750 probe.


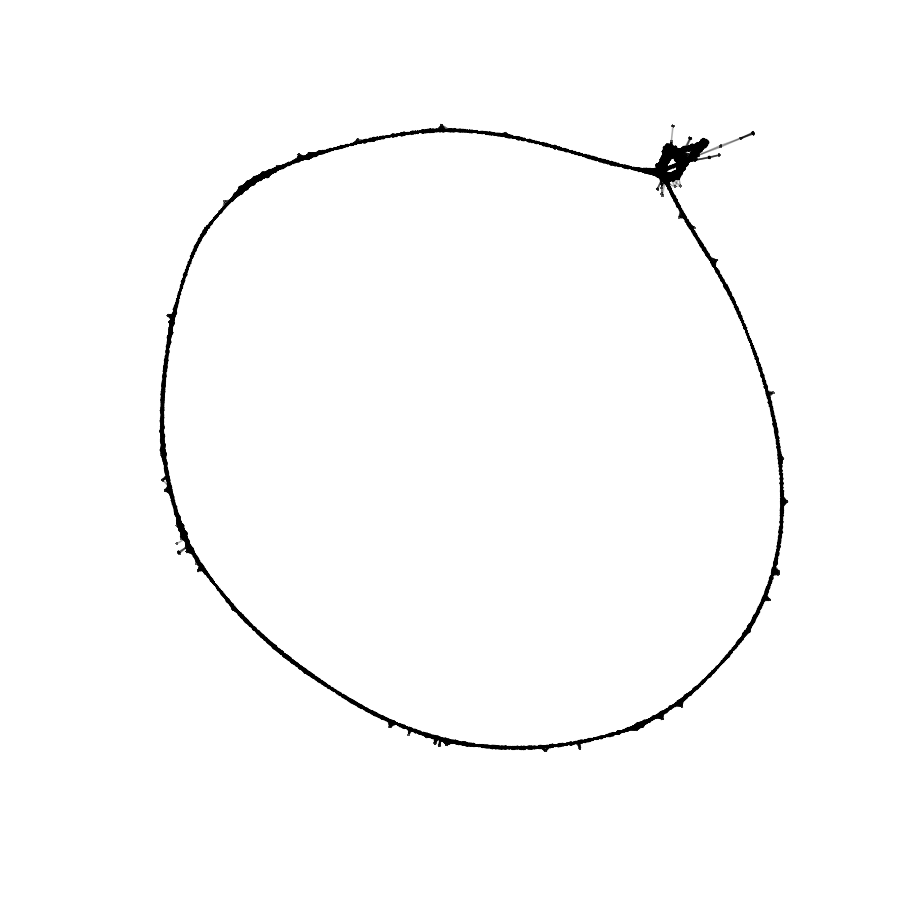


Figure S4 Results of graph-based clustering for the 45S rDNA cluster.


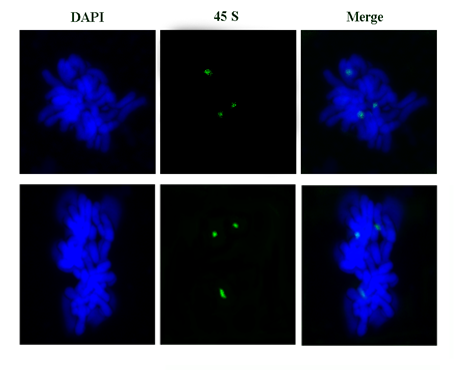


Figure S5 Additional figures showed there are only three 45S rDNA loci in this *Allium* *cepa* cultivar.

Figure S6 Co-clustering with 600000 randomly selected reads from Peška et al. 2019 (P) and current study (F) with RepeatExplorer.

We compared number of origin reads from two datasets in each clusters in the top 100 clusters. X axial: number of clusters. Y axial: (P-F)/(P+F).

The results suggest these two datasets are similar because both of them are onion. But there have different repeat composition in many clusters.

Supplemental data: FASTA file containing sequences with minimal 5 times read depth from 162 clusters we analyzed.

Table S1 Summary of the other repeats for FISH which yield smear signals

| Tandem repeats | Primers 5′–3′ | Accession number  in NCBI | Size of repeat unit (bp) | Expected length of PCR product (bp) |
| --- | --- | --- | --- | --- |
| AcCL77 | F:ATAggAgACTTTTCAAAAgACCg  R:ACATAACTgATATAACATCCCAgAACAA | LR699098.1 | 1312 | 1312 |
| AcCL107 | F: ACgATTTTTCCATgTAgCCC  R: AAACCTgTgCAgTTAATgCg | LR606193.1 | 521 | 496 |
